# Semi-Supervised Active Learning for Sound Classification in Hybrid Learning Environments

**DOI:** 10.1371/journal.pone.0162075

**Published:** 2016-09-14

**Authors:** Wenjing Han, Eduardo Coutinho, Huabin Ruan, Haifeng Li, Björn Schuller, Xiaojie Yu, Xuan Zhu

**Affiliations:** 1 Language Computing Lab, Samsung R&D Institute of China - Beijing (SRC-B), Beijing, China; 2 Department of Music, University of Liverpool, Liverpool, United Kingdom; 3 Department of Computing, Imperial College London, London, United Kingdom; 4 Department of Computer Science and Technology, Tsinghua University, Beijing, China; 5 School of Computer Science and Technology, Harbin Institute of Technology, Harbin, China; 6 Complex Systems Engineering, University of Passau, Passau, Germany; Ulm University, GERMANY

## Abstract

Coping with scarcity of labeled data is a common problem in sound classification tasks. Approaches for classifying sounds are commonly based on supervised learning algorithms, which require labeled data which is often scarce and leads to models that do not generalize well. In this paper, we make an efficient combination of confidence-based Active Learning and Self-Training with the aim of minimizing the need for human annotation for sound classification model training. The proposed method pre-processes the instances that are ready for labeling by calculating their classifier confidence scores, and then delivers the candidates with lower scores to human annotators, and those with high scores are automatically labeled by the machine. We demonstrate the feasibility and efficacy of this method in two practical scenarios: pool-based and stream-based processing. Extensive experimental results indicate that our approach requires significantly less labeled instances to reach the same performance in both scenarios compared to Passive Learning, Active Learning and Self-Training. A reduction of 52.2% in human labeled instances is achieved in both of the pool-based and stream-based scenarios on a sound classification task considering 16,930 sound instances.

## Introduction

Sound classification is a relatively recent topic in the audio analysis research community when compared to speech and music analysis. Yet, it has a wide range of applications such as multimedia data search, context awareness and activity detection [1–4], security surveillance [5, 6], military interest tracking [7], assistive devices for independent living [8], healthcare monitoring [9, 10], among others.

In Table 1, we show an overview of state-of-the-art research in sound classification. Noticeably, two main features characterize this area of research. Firstly, statistical classifiers and fully supervised learning algorithms are the most common approaches to sound classification. This means that large amounts of training data (typically labeled by human annotators) are required to create robust classification systems. Secondly, prototypical databases with size less than 10,000 instances are employed in most case. Indeed, and although the largest database mentioned in Table 1 comprises as many as 10,500 instances, the average size of each sound class is as small as 100 instances. In comparison with automatic speech recognition research where typical corpora comprise hundreds of hours of transcribed speech, annotated data in sound classification is scarce. Therefore, there is a gap between the desirability of sufficient labeled data for training robust models and the scarcity of annotated corpora.

**Table 1:** Overview of state-of-the-art research in sound classification. For features, BoAP: bag-of-audio-phrases descriptor, UFL: unsupervised feature learning, E: energy, SF: spectral features, ZCR: zero-crossing rate, TFB-ED: triangle filter bank and eigen-decomposition, MFCC: mel-frequency cepstral coefficients, STE: subband temporal envelopes, and for classifiers, SVM: support vector machines, RF: random forest, KFDA: kernel Fisher discriminant anlysis, HMM: hidden Markov models, for learning methods, FS: fully supervised learning.

Work	#Clips	#Classes	Features	Classifiers	Learning methods	Domains
[1]	1,479	22	BoAP	SVM	FS	human activity
[2]	8,732	10	UFL	RF	FS	urban environment
[3]	5,949	62	E+SF+ZCR	SVM	FS	surveillance
[6]	650	3	TFB-ED	KFDA	FS	environment
[9]	115/10,500	7/105	MFCC	HMM	FS	healthcare
[11]	705	10	STE	SVM	FS	canteen

While the development of web technology has allowed free access to vast amounts of sound media data for research usage, the shortage of labeled data remains an important issue that compromises the development of robust sound classification systems, which in turn limits their performance in practical scenarios [12–14]. To our best knowledge, even the largest environmental sound database ESC-US [15] so far contains only a limited number of labeled instances (2,000 instances) and a large amount of unlabeled instances (250,000 instances). This situation can be attributed to the burdensome and costly annotation process that requires assigning a predefined label to each of the various sound samples, which is especially critical for large databases [15]. Given this scenario, it is of extreme importance to develop techniques that allow the development of sound classification systems using databases with only partial human annotations available. This issue is addressed in this paper, and our proposal to overcome the above mentioned limitations is to combine Active Learning (AL) and Semi-Supervised Learning (SSL). With this approach, we target real-use scenarios whereby machines are required to make sense of the acoustic world surrounding them in meaningful ways by learning autonomously (SSL), through interacting with humans (AL), and by continuously adapting to a specific environment. Additionally, it also reduces the need for human labeled data for the development of robust sound classification systems.

### The best of two worlds: AL and SSL

AL [16] is a Machine Learning technique that aims at achieving greater accuracy with fewer training labels by (actively) choosing the data from which it learns. In contrast with the most commonly used Passive Learning (PL) techniques that randomly select instances from data pools to be labeled, AL algorithms select those instances that are the ‘most informative’ (with respect to a given measure function), and subsequently query human or machine annotator for labeling. The informativeness of the instances to be selected concerns their potential to improve the model’s performance by selecting the best examples during training. There are various strategies by which the informativeness of unlabeled samples can be processed (as detailed in the next section), and the effectiveness of AL has been shown in typical classification tasks such as automatic speech recognition [17], multimedia retrieval [18], speech emotion recognition [19], among others.

As a result of employing an certainty-based AL query strategy, especially when it comes to a large scale raw data collection, a considerable number of unlabeled instances will be left out because of their high confidence scores (i. e., low informativeness). Here, we consider to further exploit this remaining set of instances (which are not selected for the human to label) with a traditional SSL method. These instances, and their corresponding labels automatically annotated by the machine classifier, will be added to the human-labeled set to create a new, larger training set. As a result, we will combine AL and SSL methods to reduce the amount of human-labeled data. Specifically, human annotators are required to label only those instances with the lowest certainty as determined by the AL algorithm, while the remaining instances (those with the highest certainty) are automatically labeled by a machine annotator. Then, both groups of instances are fused and used to re-train the classifier. We will refer to this approach as Semi-Supervised Active Learning (SSAL) throughout this paper. The effectiveness of SSAL in reducing the amount of data to be labeled by human annotators will be validated in a sound database with a size of 16,930 instances.

The major contribution of this work is the application of a hybrid method combining AL and SSL in the field of sound classification, which is of extreme importance to the field given the scarcity of labeled data and the need to minimise the costs associated with human annotations. Furthermore, we provide a detailed operationalization of the proposed method in two target scenarios: pool-based (all data is available at once) and stream-based (a practical scenario whereby instances are gathered sequentially from actual distributions) scenarios.

## Related work

### Active Learning

One of the most promising approaches proposed in the literature to efficiently exploit unlabeled data for model development is AL [20–22]. By estimating the informativeness of the unlabeled instances, AL selects only those with high potential to improve the model’s performance for annotation. There are various strategies by which such informativeness can be processed (aka, *query strategies*), and, according to the different types of feedback considered, at least three categories can be generalized from previous work [16]: 1) *certainty-based sampling*, 2) *query-by-committee*, 3) *expected error reduction*. In the first type of strategy, the model (or active learner) determines the certainty of the predictions on unlabeled data based on a previously trained model, and queries an annotator for the labeling of those with the least certain classification. This is perhaps the most commonly used query strategy. For instance, it has been applied in text classification [22], automatic speech recognition [17], speech emotion classification [19], audio retrieval [23], among others. The second type of strategy (*query-by-committee*) involves two or more classifiers and the selection of those instances about which the various models disagree the most, which are then delivered for human annotation. This strategy can also be employed in regression tasks by measuring disagreement as the variance among the committee members [24]. The third type of strategy (*expected error reduction*) is a decision-theoretic approach that aims to estimate how much the model’s generalization error is likely to be reduced. The instances estimated to have a high impact on the expected model’s error are selected for human annotation. This strategy has been adopted for text classification task with Naive Bayes models [25], and leads to a dramatic improvement over *certainty-based* and *query-by-committee* strategies. Unfortunately, the expected *error reduction method* is also, in most cases, the most computationally expensive [16]. The effectiveness of AL and the various query strategies has been shown in typical classification tasks [16, 19, 22–25].

### Semi-Supervised Learning

Similarly to AL, the goal of SSL techniques is to exploit the availability of unlabeled data for model training and improvement. Two broad categories of SSL have been investigated to date: *self-training* [26] and *co-training* [27, 28]. *Self-training* is a technique that permits to automatically annotate unlabeled data by using a preexisting model trained on a smaller set of labeled data. Usually, those instances of the unlabeled data set that are predicted with the highest degree of confidences are added to the training set (together with the respective labels), and the classifier is re-trained with the new (larger) set. This procedure is then repeated iteratively until a certain target performance is achieved (or until no more unlabeled candidate data is available). This approach is very attractive and useful to enhance the robustness of existing classifiers, because it does not require the intervention of human annotators [29, 30]. The effectiveness of *self-training* has been demonstrated in various areas, including spoken language understanding [31], handwritten digit and text classification [32], and sound event classification [33].

Another set of algorithms with the potential to exploit unlabeled data pools is *multi-view learning* [30, 34, 35]. *Multi-view learning* techniques focus on improving the learning process by training different models for the same task concurrently, but using different feature sets (aka, “views”) [16]. *Co-training* is one of the earliest schemes for *multi-view learning* proposed in the literature. In this method, two models are initially trained with two distinct different feature sets of the same labeled data set. Then, the most confident predictions of each model on the unlabeled data are added to the training set to train each other. The algorithm relies on three assumptions or conditions: (a) *sufficiency*: each “view” is sufficient for classification on its own, (b) *compatibility*: the target functions in both “views” predict the same labels for co-occurring features with high probability, and (c) *conditional independence*: the “views” are conditionally independent given the class label [27].

### Combining Active and Semi-Supervised Learning

AL strategies can greatly reduce the time-consuming and expensive human labeling work and lead to excellent performance improvements [16]. Nevertheless, AL is still inadequate for some situations in which obtaining a large amount of human annotations is unpractical (or not possible at all), and therefore needs to be minimized. Given that SSL also aims at using unlabeled data in an efficient way, but without the intervention of human annotators, it is natural to think about combining both techniques. Indeed, various examples can be found in the literature and are summarized in Table 2. One of the first works exploring combinations of AL and SSL algorithms was reported in [36]. Later, [34] proposed a variant of *query-by-committee* method, which is known as *co-testing*. In this method, two classifiers were trained separately on two different views (similarly to *co-training*), and the unlabeled instances in which the classifier disagree the most (‘contention points’) were selected for human annotation. *Co-testing* was then combined with *co-training* using an *expectation maximization* (co-EM) algorithm to automatically label instances that showed a low disagreement between the two classifiers. The combined method proposed in [34] clearly outperformed co-EM, general *co-testing* and *co-training* in Web pages and pictures classification. [37] also achieved significant performance improvements by combining *co-testing* and *co-training* methods in image retrieval compared to either *co-testing* or *co-training* retrieval method. Certainty-based AL has been also used alongside *self-training* to significantly reduce the human labeling effort in spoken language understanding [31] and natural language processing [38]. In the work presented in this paper, we will tandem certainty-based AL and *self-training* methods for sound classification.

**Table 2:** Overview of previous work combining Active and Semi-Supervised Learning techniques, and the work proposed in this paper. AL: Active Learning, SSL: Semi-Supervised Learning, QBC: Query-By-Committee, EM: Expectation Maximization, SBC: Similarity-based Classifier, CRFs: Conditional Random Fields, SVM: Support Vector Machines.

Article	AL method	SSL method	Scenario	Classifier	Domain	Year
[36]	QBC	EM	pool	naive Bayes	text classification	1998
[34]	Co-Testing	Co-EM	pool	naive Bayes	Web pages & pictures classification.	2002
[37]	Co-Testing	Co-Training	pool	SBC	content-based image retrieval	2004
[31]	Certainty-based	Self-training	fixed & dynamic pool	Boosting	spoken language understanding	2005
[38]	Certainty-based	Self-training	stream	CRFs	natural language processing	2009
this work	Certainty-based	Self-training	pool & stream	SVM	sound classification	2015

## Active Learning in two scenarios

In this paper we adopt an certainty-based AL approach. Moreover, we consider two target scenarios: pool-based scenario and stream-based scenario. The focus on the first scenario tackles situations where a large pool of unlabeled data can be gathered at once (the most common in previous work; cf. Table 2). In this case, before deciding which instances should be selected in each training iteration, every instance in the pool can be evaluated in terms of their informativeness. The second scenario fits a practical scenario in which unlabeled instances are gathered sequentially from actual distributions (e.g., an online sound processing system). In this case, the (active) learner decides whether to keep or discard each instance individually. Unlike the pool-based scenario, the stream-based scheme is more appropriate for situations in which memory or processing power may be limited (e.g., mobile and embedded devices) [16].

A detailed description of the AL strategies used in this paper are shown in Tables 3 and 4. In both strategies we start with a small set of labeled instances *S*_*l*_ for training an initial classifier *M*. With this classifier, we estimate the confidence scores *C*s for the instances that are candidates for labeling. In the pool-based scenario, the entire pool of unlabeled instances *S*_*u*_ is estimated, and only those instances with confidence scores equal to or lower than the pre-defined threshold *th*_*a*_ are selected for human annotation. In the stream-based scenario, the instances are analyzed sequentially and selections are made individually. At each iteration, the buffer *B* is send to human for annotation as soon as it is full filled with instances with confidence scores less than the pre-defined threshold *th*_*a*_. The threshold *th*_*a*_ is determined by the human labeling resources available or by the performance of the current classifier.

**Table 3:** Certainty-based Active Learning algorithm in a pool-based scenario.

**Input:**
*S*_*l*_: a small set of labeled instances
*S*_*u*_: a large pool of unlabeled instances
*M*: an initial classifier trained on *S*_*l*_
*th*_*a*_: the confidence threshold
**Do:**
Classify each instance in *S*_*u*_ using classifier *M* and calculate the confidence score *C* for each selected instance.
Select those instances with *C*s that are equal to or lower than threshold *th*_*a*_, and submit them to human annotation.
Refer to the new labeled set as *S*_*new*_.
**S**_*l*_ = *S*_*l*_ ∪ *S*_*new*_, *S*_*u*_ = *S*_*u*_ − *S*_*new*_.
Re-train classifier *M* using new *S*_*l*_.
**Until** *S*_*u*_ = ∅/labeler is unavailable/model training converges

**Table 4:** Certainty-based Active Learning algorithm in a stream-based scenario.

**Input:**
*S*_*l*_: a small set of labeled instances
*S*_*u*_: a large stream of unlabeled instances
*M*: an initial classifier trained by *S*_*l*_
*B*: a fixed buffer
*th*_*a*_: the confidence threshold
**Do**
Classify current instance from *S*_*u*_ using classifier *M* and calculate the confidence score *C*.
**if** *C* < *th*_*a*_
Retain current instance in buffer *B*.
**otherwise**
Discard current instance.
**end if**
**if** buffer *B* is full
Submit instances in *B* to human annotation.
Refer to the new labeled set as *S*_*new*_.
*S*_*l*_ = *S*_*l*_ ∪ *S*_*new*_, *S*_*u*_ = *S*_*u*_ − *S*_*new*_.
Re-train classifier *M* using new *S*_*l*_.
**end if**
**Until** *S*_*u*_ is interrupted/labeler is unavailable/model training converges

## Semi-supervised Learning

As mention, in order to further reduce the need for human annotation and enhancing the classification performance, we complement the AL phase with *self-training*. A detailed description of this strategy is presented in Table 5. First, we train an initial model *M* using an initial (small) set of human-labeled data *S*_*l*_. Then, we classify the unlabeled instances *S*_*u*_ and calculate the confidence scores (as it will be defined later in this paper). Finally, we select those unlabeled instances with confidence scores equal to or greater than a given threshold *th*_*s*_, and add them (together with the respective machine-annotated labels) to the training set for the next iteration.

**Table 5:** Semi-Supervised Learning strategy.

**Input:**
*S*_*l*_: a small set of labeled instances
*S*_*u*_: a large pool of unlabeled instances
*M*: an initial classifier trained by *S*_*l*_
*th*_*s*_: the confidence threshold
**Do:**
Classify every instance in *S*_*u*_ using classifier *M* and calculate the corresponding confidence score *C*.
Select those instances with *C*s that are equal to or higher than threshold *th*_*s*_, and label them with corresponding predicted categories.
Refer to the machine-labeled set as *S*_*new*_.
**S**_*l*_ = *S*_*l*_ ∪ *S*_*new*_, *S*_*u*_ = *S*_*u*_ − *S*_*new*_.
Re-train classifier *M* using the new set *S*_*l*_.
**Until** model training converges/unlabeled data is unavailable

There are two parameters that need to be set in this strategy: the confidence threshold *th*_*s*_ and the size of the initial human-labeled data set |*S*_*l*_|. Regarding the first, which defines the amount of unlabeled data to be selected at each iteration of the algorithm, we have to find a compromise between the impact of adding noisy instances (low *th*_*s*_) and adding less informative ones (high *th*_*s*_). Regarding the second, we have to consider that if the set is too small the initial model will have a high classification error rate, and if the set is too large no improvement over the initial model can be expected because there is nothing to be learned. In this paper, we will optimize these parameters as it will be described in experimental section.

## Combining Active and Semi-supervised Learning

As discussed above, active and semi-supervised learning share the common goal to reduce the amount of human annotation effort by means of selective data sampling. However, they further share the same criteria for data sampling—the confidence score. The difference is that they achieve their goals from opposite ‘ends’: active learning samples data with low classifier confidence, while semi-supervised learning samples the data with high confidence. Thus, it comes naturally to combine them for more efficient model learning. Our proposed approach is as follows.

By using two given confidence thresholds *th*_*ssaL*_ and *th*_*ssaH*_, the candidate instances that are evaluated for labeling can be sampled to generate two subsets: one subset containing instances whose confidence scores are lower than *th*_*ssaL*_, and another subset containing those instances whose confidence scores are equal to or higher than *th*_*ssaH*_. It follows that the former subset of instances is selected for human labeling, and the latter for machine labeling. This approach can be referred to as *Semi*-*Supervised* *Active* *Learning* (SSAL), since it tandems the standard fully supervised AL strategy with a bootstrapping strategy SSL, (i.e., *self-training*). SSAL is formally described in Tables 6 and 7 for pool-based and stream-based scenarios, respectively.

**Table 6:** Semi-Supervised Active Learning in a pool-based scenario.

**Input:**
*S*_*l*_: small set of labeled instances
*S*_*u*_: large pool of unlabeled instances
*M*: initial classifier trained by *S*_*l*_
*th*_*ssaL*_, *th*_*ssaH*_: confidence thresholds
**Do:**
Classify every instance in *S*_*u*_ using classifier *M* and calculate the corresponding confidence score *C*.
Select instances with *C*s lower than *th*_*ssaL*_ from *S*_*u*_ and submit them to human annotation.
Refer to the new labeled set as $S_{new}^{a}$.
$S_{l}=S_{l}\cup S_{new}^{a},\;S_{u}=S_{u}-S_{new}^{a}.$
*Re-train the classifier *M* using the new *S*_*l*_.
Select those instances with *C*s equal to or higher than *th*_*ssaH*_, and add the corresponding predicted labels.
Refer to the machine-labeled set as $S_{new}^{s}$.
$S_{l}=S_{l}\cup S_{new}^{s},\;S_{u}=S_{u}-S_{new}^{s}.$
*Re-train the classifier *M* using the new *S*_*l*_.
**Until** *S*_*u*_ = ∅/labeler is unavailable/model training converges

* Note that the model is re-trained twice at each learning iteration.

**Table 7:** Semi-Supervised Active Learning in a stream-based scenario.

**Input:**
*S*_*l*_: small set of labeled instances
*S*_*u*_: large stream of unlabeled instances
*M*: initial classifier trained by *S*_*l*_
*B*: fixed buffer
*th*_*ssaL*_, *th*_*ssaH*_: confidence thresholds
**Do**
Classify current instance from *S*_*u*_ using classifier *M* and calculate its confidence score *C*.
Retain current instance in buffer *B*.
**if** Buffer *B* is full
**S**elect those instances with *C*s lower than *th*_*ssaL*_ from *B* and submit them to human annotation.
Refer to the human-labeled set as $S_{new}^{a}$.
$S_{l}=S_{l}\cup S_{new}^{a},\;S_{u}=S_{u}-S_{new}^{a}$
*Re-train classifier *M* using the new set *S*_*l*_, and re-classify the remaining instances in *B*.
Automatically label those instances with *C*s higher than *th*_*ssaH*_ in *B* with predicted labels.
Refer to the machine-labeled set as $S_{new}^{s}$.
$S_{l}=S_{l}\cup S_{new}^{s},\;S_{u}=S_{u}-S_{new}^{s}.$
*Re-train the classifier *M* using the new *S*_*l*_.
**end if**
**Until** *S*_*u*_ is interrupted/labeler is unavailable/model training converges

* Note that the model is re-trained twice at each learning iteration.

In the pool-based scenario, at every learning iteration, we incrementally increase the initial training set with a set of human-labeled instances (those with confidence scores lower than the threshold *th*_*ssaL*_), and a variable number of machine-labeled instances (those with confidence scores equal to or higher than the threshold *th*_*ssaH*_. As can be observed from Table 6, there are twice as many model re-training operations in each learning iteration compared to the individual AL and *self-training* approaches. In our approach, we first re-train the model with the human-labeled date set $S_{new}^{a}$ (AL phase), and then produce the machine-labeled data set $S_{new}^{s}$ (SSL phase). The purpose of this design aims at improving the quality of the data set $S_{new}^{s}$ by making use of a model previously trained with reliable (human) labels. This is very important for the SSL phase, since having the model trained first with reliable annotations from the AL phase will decrease the amount of noisy data (instances with potentially wrong labels assigned). This will avoid the deterioration of the performance that can occur in the SSL phase. The same approach for avoiding noisy data is adopted in the stream-based scenario, see Table 7. Additionally, we continuously fill the buffer *B* with new instances. Once the buffer is full, two confidence thresholds *th*_*ssaL*_ and *th*_*ssaH*_ are adopted for data splitting.

## Database and Acoustic Features

For the purpose of this work, we use the FindSounds database (http://www.findsounds.com/types.html—accessed on 25 July 2011), which provides a large amount of varied real life sounds already categorized. In order to better suit our study and avoid very unbalanced class distributions, we discarded those categories with only a few instances (insects, with 7 subsets, and holidays, with 5 subsets) and combined “birds” and “animals” categories in to a single category (“Animals”). The database used in this study comprises seven categories (out of sixteen) of sounds: 1) **People**: sounds resultant from 45 different human behaviors, such as coughing, laughing, moaning, kissing, baby’s cry; 2) **Animals**: sounds from 69 different non-bird animals (e.g., cat, frog, bear, lamb, blackbird) and 16 kinds of birds. 3) **Nature**: 19 kinds of nature sounds (e.g. earthquake, ocean waves, flame, rain, wind); 4) **Vehicles**: sounds produced by 34 different types of vehicles (e.g., car, motorbike, helicopter) and related actions (e.g., braking, closing door); 5) **Noisemakers**: comprising 13 types of sound events (e.g., alarm, bell, whistle, horn); 6) **Office**: original office space sound events (e.g, typing, printing, phone calls, mouse clicking) 7) **Musical Instruments**: sounds from 62 different musical acoustic and electronic instruments (e.g., bass, drum, synthesizer).

In total, there are 16,930 sound instances in our database with durations ranging from 1 to 10 seconds, which correspond to (approximately) 15 hours of environmental sounds. All sound files were converted into raw 16 bit encoding, mono-channel, and 16 kHz sampling rate, as various formats and rates were used in the original versions retrieved from the web. The details of the database and categories used are shown in Table 8. Throughout this paper we will refer to the database as FINDSOUNDS. (The whole database together with corresponding labels can be downloaded for research and academic purpose from https://www.dropbox.com/sh/nmw4ef7ma5ok8df/AACnx63TtkrwXyHyiJ0FpSw8a?dl=0.)

**Table 8:** Description of the subset of the FindSounds database used in this paper.

Category	# Subsets	# Clips	Duration [h]
**People**	45	2,540	2 h 09 min
**Animals**	85	2,834	2 h 42 min
**Nature**	19	937	1 h 17 min
**Vehicles**	34	2,166	2 h 47 min
**Noisemakers**	13	2,010	1 h 56 min
**Office**	18	1,769	1 h 01 min
**Musical Instruments**	62	4,674	3 h 49 min
**Total**	**276**	**16,930**	**15 h 41 min**

In order to evaluate the effectiveness of the new method proposed in this paper, we adopted the baseline audio feature set used in the Audio/Visual Emotion Challenge (AVEC) 2012. This feature set comprises 1,841 features that result from a systematic combination 25 energy- and spectral-related low-level descriptors (LLDs) with 42 functionals, 6 voicing-related LLDs with 32 functionals, 25 delta coefficients of energy/spectral-related LLDs with 23 functionals, 6 delta coefficients of voicing-related LLDs with 19 functionals, and 10 voiced/unvoiced durational features (for full details on the feature set please refer to [39]). All features and functionals were extracted with the OpenSMILE toolkit [40].

## Experiments and Results

In this section, we describe a series of experiments conducted with the purpose of empirically investigating the effectiveness of three learning methods in the context of sound classification: 1) certainty-based AL; 2) SSL; and 3) our proposed method, SSAL.

### Experimental Setup

For every experiment presented in this paper, we run a 10-fold cross validation (the split is 90% for train, 10% for test) to obtain stable estimates of the algorithm’s performance. We compute unweighted average recalls (UARs), the sum of the accuracies per class divided by the number of classes without considerations of instances per class, as evaluation metric. For result representation in figures below, the UARs over 10 rounds along with the standard deviation bar are used. All experiments use the FINDSOUNDS corpus introduced in previous section. In order to deal with the imbalance between the number of instances in each category (or class distributions), we employ data oversampling in the training set in order to add more instances belonging to the less represented classes. Oversampling is performed in WEKA [41] using the Synthetic Minority Over-sampling Technique (SMOTE) [42] (WEKA defaults settings are used).

Specifically, SMOTE does oversampling by creating “synthetic” examples for minority class. It takes each minority class sample and produces synthetic examples making use of all of the *k* minority class nearest neighbors. Depending upon the amount of oversampling required, neighbors from the *k* nearest neighbors are randomly chosen. Our experimental setup currently uses 5 nearest neighbors. Synthetic samples are generated in the following way: Take the difference between the feature vector (sample) under consideration and its nearest neighbor. Multiply this difference by a random number between 0 and 1, and add it to the feature vector under consideration. This approach effectively forces the decision region of the minority class to become more general.

As classifier we use Support Vector Machines (SVM) [43] with linear kernels and pairwise multi-class discrimination sequential minimal optimization (implemented in the WEKA framework [41]). SVMs are supervised learning models based on the concept of decision hyperplanes that define decision boundaries—hyperplanes in a multidimensional space that separate sets of elements based on class memberships. The output value of SVMs is the distance of a specific point from the separating hyperplane, but a central aspect of our AL approach is the calculation of the confidence scores. To convert these distances to probability estimates within the range of [0, 1] there are various parametric and nonparametric approaches. In this work, we employed a parametric method of logistic regression proposed in [44], which is one of the most frequently used approaches to transform the output distances of SVMs into (pseudo) probabilistic values [23, 45, 46]. This method assumes that the posterior probability consists of finding the parameters *A* and *B* for a form of sigmoid function:

$$\begin{array}{c} P\left(y|f\left(x\right)\right)=\frac{1}{1+\text{exp}(Af(x)+B)}, \end{array}$$ (1)

mapping the value *f*(*x*) into probability estimates *P*(*y*|*f*(*x*)). For each instance, the sum of the posterior probability for all classes is equal to 1. This probability indicates the classifier’s confidence about the predicted label given. We then define the confidence score of *x* as follows:

$$\begin{array}{c} C(x)=P(y|f(x)). \end{array}$$ (2)

Additionally, in the context of pool-based AL, and AL phase in SSAL experiments, instead of using a threshold mechanism for data splitting as described in Tables 3, 6 and 7, we select 500 instances with lowest confidence scores for human annotation in each learning round. And for stream-based AL as described in Table 4, we set the instances buffer size as 500 for the sake of consistency. The reason behind is to fix the number of human labeled instances in each learning iteration to further make an unified performance comparison platform for different learning methods.

### Confidence Scores Evaluation and Distribution

The learning methods proposed in this paper are based on two assumptions. First, the confidence scores (cf. Eq (2)) are good indicators of the classifier’s output certainty level. This is essential to ensure that the instances with the lowest classification certainty (low confidence scores) are selected to be delivered for human annotation, and the instances with high classification certainty (high confidence scores) are directly added to training data set with labels automatically given by the machine annotator. Second, only a small portion of the unlabeled instances are classified with low certainty, otherwise human effort cannot be dramatically reduced.

**Fig 1:**
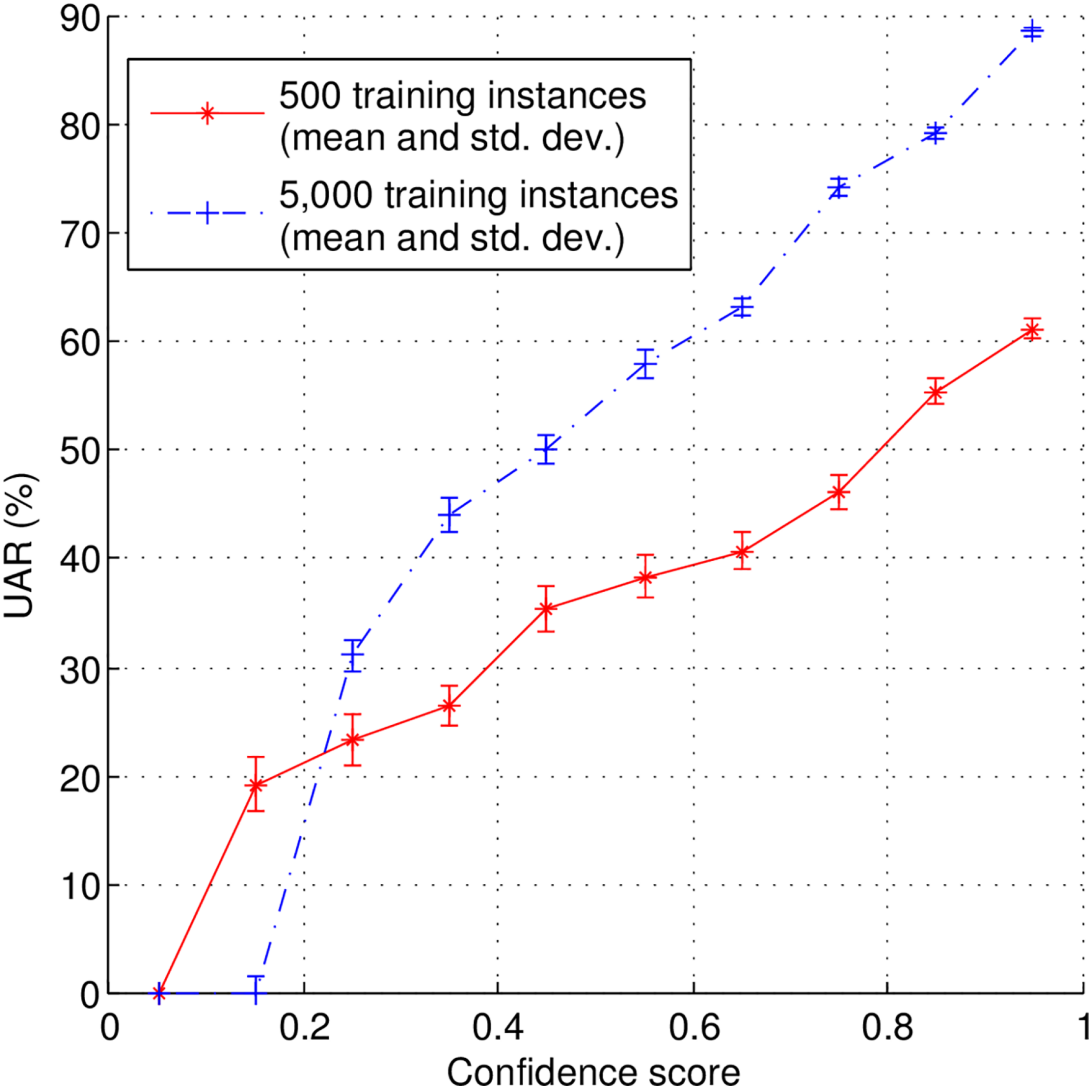
Relationship between classifier’s classification UARs and confidence scores for 500 and 5,000 initial training instances.

**Fig 2:**
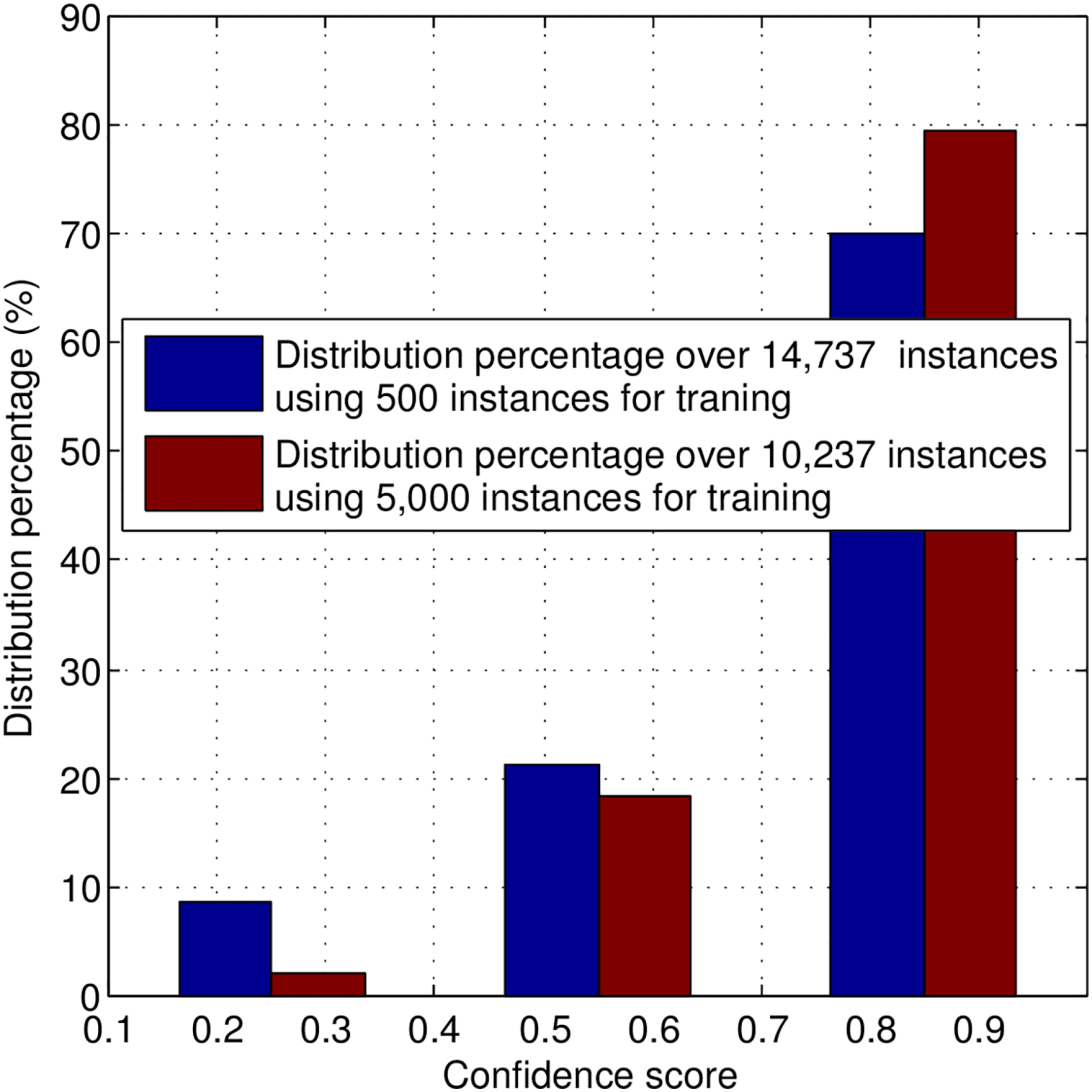
Distribution percentage of classifier confidence scores for 500 (blue) and 5,000 (red) training instances. (There is no instance assigned with confidence score falling in the range of [0.0, 0.1].)

Before starting our experiments, it is relevant to evaluate whether these two assumptions are in fact supported. To do so, we train a SVM classifier with 500 and 5,000 instances (randomly selected from a training set considering class balance), and test it on the remaining (unlabeled) instances (14,737 and 10,237, respectively). In Fig 1, we show the relation between the test instances’ confidence scores and corresponding UARs, and in Fig 2, we show the distribution of the confidence scores falling in different ranges (i.e., [0.1, 0.4), [0.4, 0.7), [0.7, 1.0]) over unlabeled instances. As it can be seen in Fig 1, an increase in the UAR of the classifier is matched by an increase in the confidence scores. Moreover, when the classifier is trained with more labeled instances, the confidence scores tend to reflect better the classifier’s UAR. Hence, the classifier confidence scores seem to reflect well the classifier’s certainty level regarding the corresponding classification results. In relation to the second assumption, as shown in Fig 2, the majority of unlabeled instances are classified with high confidence values. It is also evident that the classifier initially trained with more labeled instances, tends to classify more unlabeled instances with higher confidence levels. Therefore, only a small portion of the unlabeled data is classified with low certainty.

### Active Learning Experiments

**Fig 3:**
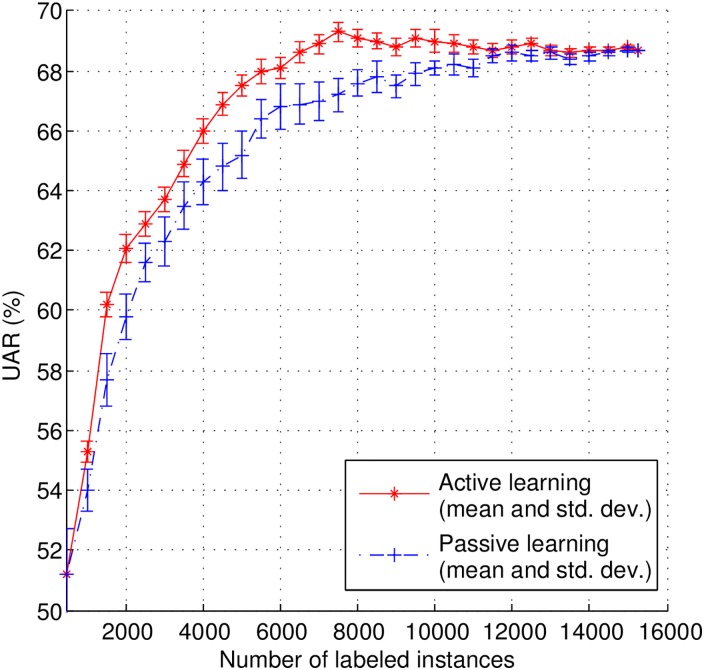
Learning curves for using active and passive learning method in pool-based scenario.

In the certainty-based AL pool-based scenario, we use the same set of 500 samples as pre-selected in above section to train the initial classifier. Then, in order to study the evolution of classification performance, we incrementally select, and manually label, 500 instances per iteration from the pool of remaining data (14,737 instances) for model re-training until all data is labeled. The learning curves (UAR vs. number of instances added) for the AL method are shown in Fig 3. Additionally, we also show the results for a passive learning (PL) method (i.e., randomly select instances for labeling) for the sake of comparison.

As it can be observed from Fig 3, the AL method effectively reduces the amount of human annotations needed to achieve a given UAR. For instance, the PL method achieves a top classification UAR up to statistical significance of 68.5% when using 11,500 instances (75.5% of the total number of instances in the data pool), while the AL approach reaches the same UAR with 43.5% less labeled data (6,500 instances). The best UAR up to statistical significance with AL, 69.3%, is achieved with only 7,500 manually labeled instances (49.2% of the total number of instances in the data pool), which is statistical significantly higher than that of PL with *p*-value = 0.0326 for two sample Kolmogorov-Smirnov test.

**Fig 4:**
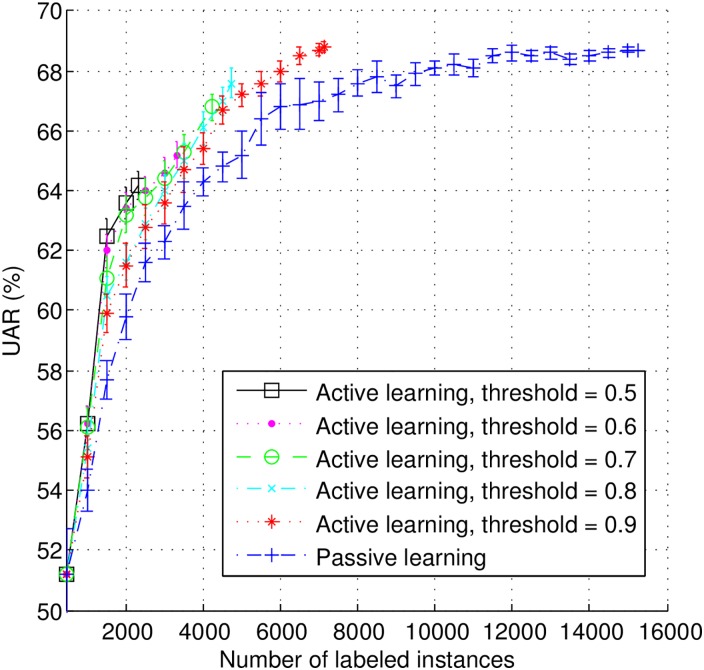
Learning curves for using active and passive learning method in stream-based scenario.

In order to simulate the stream-based scenario, we continuously sample instances from the candidate set, one by one, in a random fashion. We decide to accept or discard the selected instance immediately after sampling. Those with confidence scores lower than the given threshold are accepted and added to the buffer. As soon as the buffer is full (500 instances), the selected instances are delivered to human annotation, and finally added to the training data set (together with respective label). The model is then re-trained and the same process repeated. However, in most cases, the buffer can not be filled up in last iteration. The selected instances are still manually labeled by human for model training. Based on the analysis of the confidence score distribution shown earlier in Fig 2, which shows that only a few instances fall in the interval between 0.0 and 0.4, we decided to test five different thresholds *th*_*a*_s: 0.5, 0.6, 0.7, 0.8, and 0.9. Additionally, for the sake of comparison, we also tested the PL method, whereby instances are randomly selected (which can be considered as a stream-based AL process with 1.0 as confidence threshold). The results are shown in Fig 4.

From Fig 4, we can see that the AL approach with any of the five threshold levels leads to better classification performances with a smaller amount of labeled instances (compared to the PL approach). Furthermore, AL with lower threshold performs better than with higher threshold, which indicates that selecting instances that are more informative can lead to better performance with less annotation effort. However, lower threshold also means a larger amount of discarded unlabeled instances, which is why the learning curves with lower thresholds stop earlier—less instances are used for training. Therefore, the value of threshold should carefully be tuned according to the specific application. Quantitatively, in the best case scenario, to achieve the top classification UAR up to statistical significance of PL (68.5%, with 11,500 instances labeled), the AL method with a threshold of 0.9 requires only 6,500 instances to be annotated (43.5% less than PL). Therefore, AL efficiently reduces the need for human annotations while achieving the same performance as PL.

### Semi-supervised Learning Experiments

In this section, we evaluate the SSL method described in Table 5. Four initial training data sizes (i.e., 500, 1,000, 2,000, and 5,000) and six thresholds *th*_*s*_s (i.e., 0.6, 0.7, 0.8, 0.9, 0.95, and 1.0) are considered here. Note that with a threshold of 1.0, no machine-labeled instances are added to the initial training data set. Additionally, in each case, those learning iterations are going on until no more unlabeled data is available.

**Fig 5:**
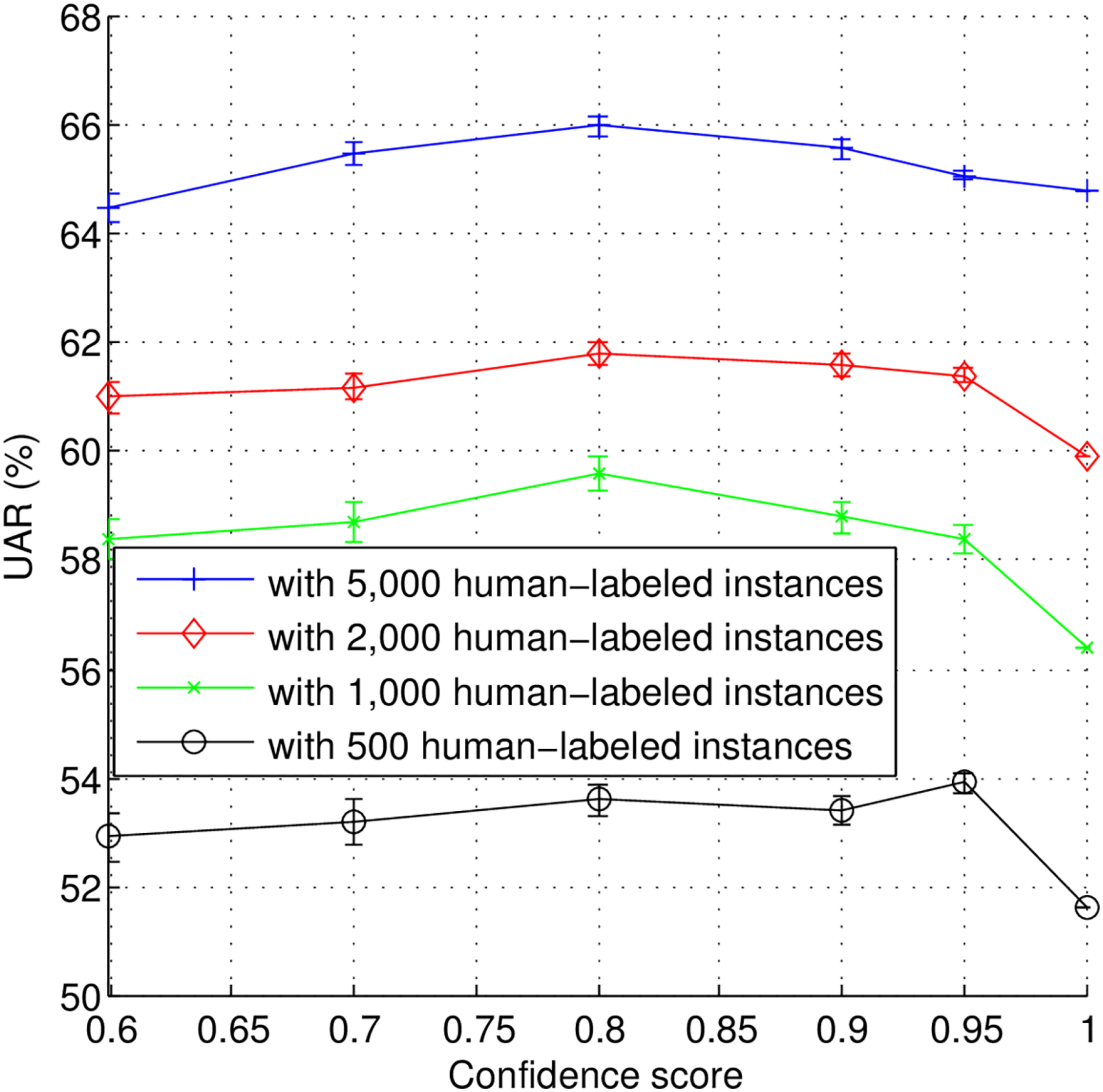
Semi-supervised learning results for varying sizes of the initial training set (different number of human labeled instances) in combination with different confidence thresholds.

The classification UAR figures for the different tests are depicted in Fig 5. As it can be seen, the best UAR with 500 human-labeled instances is achieved with a threshold of 0.95, while for other initial numbers of instances used the best UARs are achieved with a threshold of 0.8. This result may indicate that using less data to train the initial classifier may require a higher confidence threshold in order to guarantee the quality of machine labeling. With more data to train the initial classifier, the UAR of the classifier is likely to increase and lower confidence thresholds seem to ensure the informativeness of the instances.

### Semi-supervised Active Learning Experiments

The effectiveness of active and semi-supervised learning methods has been separately evaluated in the previous two sections. Both methods showed advantages in boosting the initial classification performance, while reducing manual labeling effort. In this section, we focus on assessing the combination of the two learning methods—the new method proposed in this paper—for both pool-based and stream-based scenarios.

**Fig 6:**
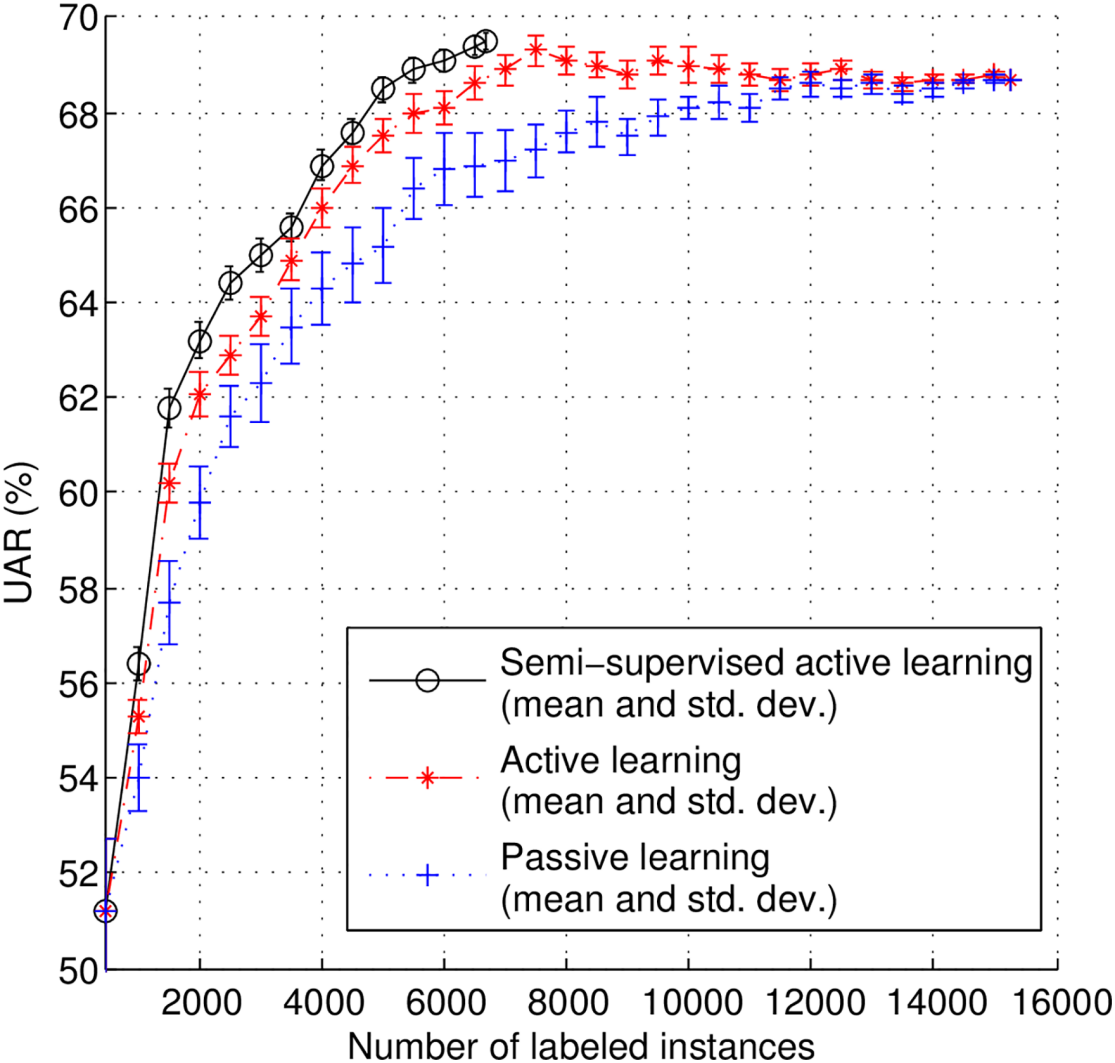
Learning curves for semi-supervised active learning (in each round 500 instances with lowest confidence scores are selected for human annotation and a variable number of instances with confidence scores above the threshold 0.95 are selected for machine annotation), active learning, and passive learning in the pool-based scenario.

In the pool-based scenario, we use the same 500 instances as in previous active learning experiments for initial model training, and then incrementally select new instances from the remaining pool (14,737 instances) for either human or machine annotation. Specifically, in each round 500 instances are selected for human labeling and a variable number of instances with confidence scores above a given threshold are selected for machine labeling. In last iteration, once less than 500 instances are available for selecting, human annotators label them all for model re-training. Fig 6 shows the classification performance of the SSAL method with a threshold of 0.95, as well as that of the AL and PL methods. As it can be observed in Fig 6, the SSAL method achieves similar classification UAR with AL (69.4% (SSAL) vs 69.3% (AL)), and outperforms the PL by circa 0.9% (69.4% (SSAL) vs 68.5% (PL)) with *p*-value = 0.0173 for two-sample Kolmogorov-Smirnov test. Moreover, the classification performance curve for SSAL stops earlier than other two since a larger amount of instances are labeled at each iteration. In order to achieve the best performance of the PL method (68.5%; 11,500 human labeled instances), SSAL requires only 5,500 human labeled instances, 52.2% less than PL and 15.4% than AL (6,500).

**Fig 7:**
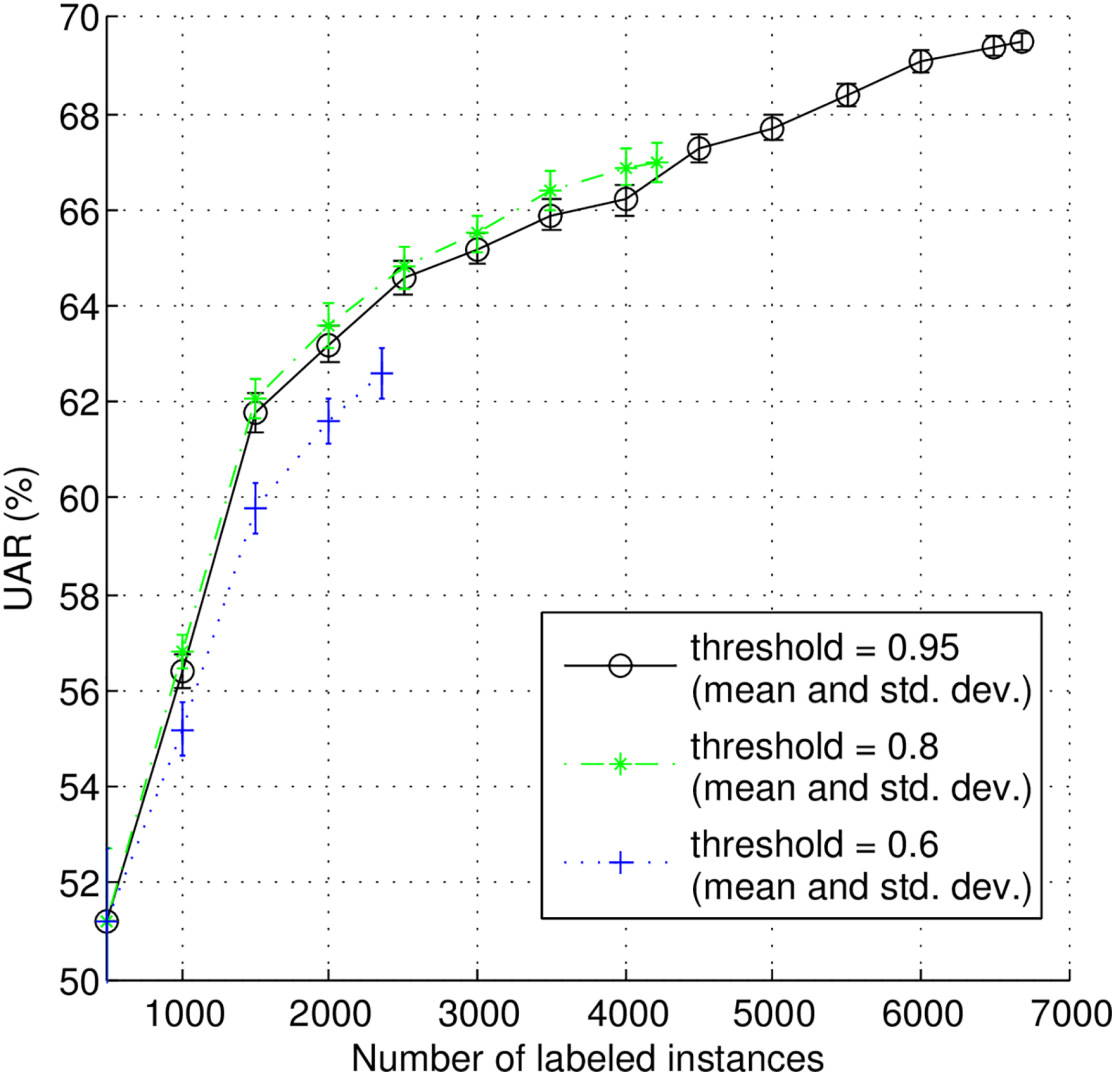
Learning curves for semi-supervised active learning with different thresholds in pool-based scenario.

In order to evaluate the impact of the confidence thresholds on SSAL in the pool-based scenario, we tested three values: 0.60, 0.80, and 0.95. The results are shown in Fig 7. With a threshold of 0.60 many selected instances are labeled by machine and the classification performance is worst compare to other two cases. A threshold of 0.80 leads to a similar classification performance curve to that of 0.95, but its curve stops earlier with lower performance level for more instances are delivered to machine for annotation. Therefore, a threshold of 0.95 is preferred in our experiments. Furthermore, these tests indicate that the tuning of the threshold level is critical for the optimization of the learning process.

In relation to the stream-based scenario, we started once more with 500 instances for the training of the initial model. In order to simulate a steady stream of incoming data, we randomly sampled new instances from the remaining set (14,737 instances) until the buffer was full (1,000 instances) in a sequential process. At this point, we selected the 500 instances with lowest confidence scores for human annotation, and the 100 instances with the highest confidence scores for machine annotation.

**Fig 8:**
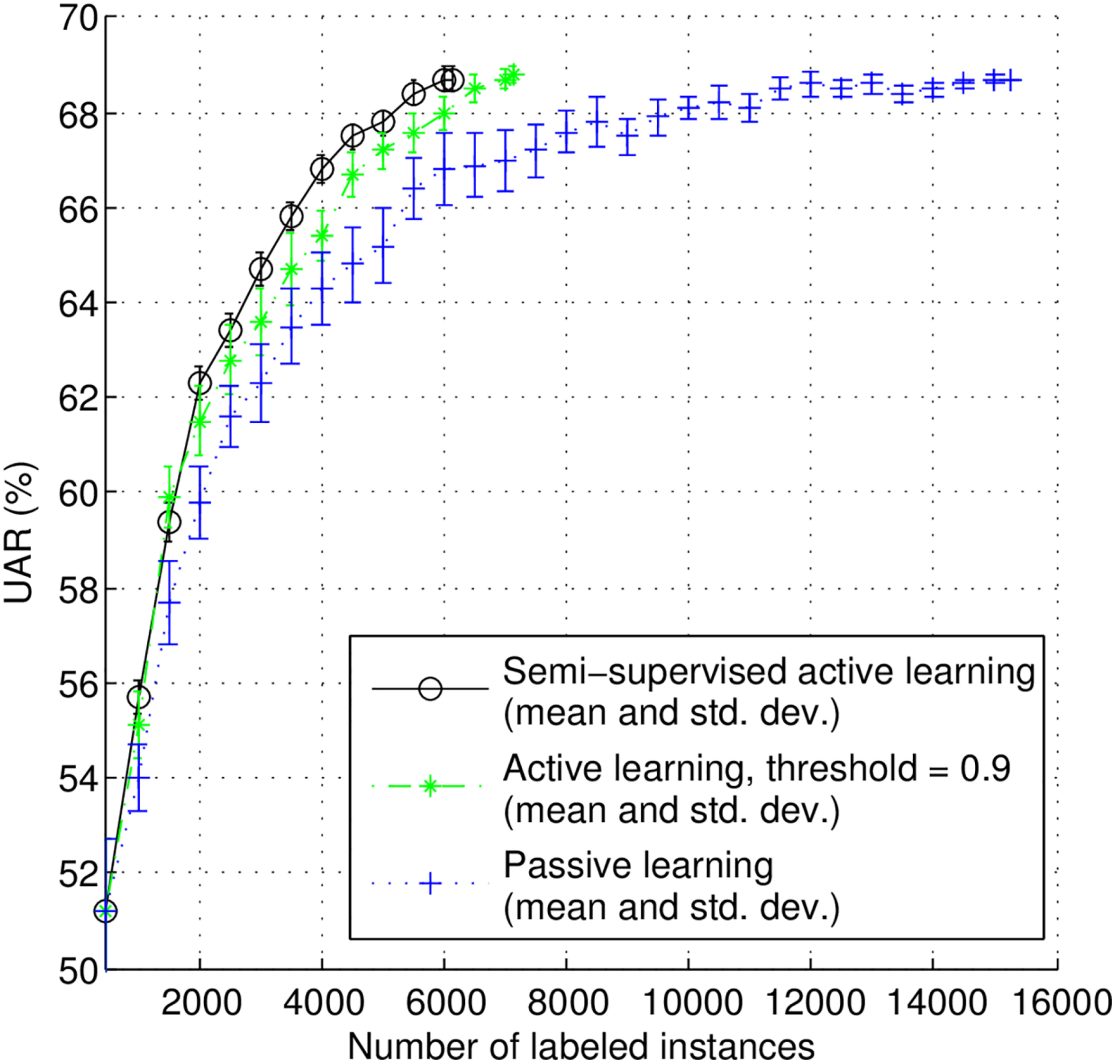
Learning curves for semi-supervised active learning (in each round 500 instances with lowest confidence scores are selected for human annotation, and 100 instances with the highest confidence scores are selected for machine annotation), active learning, and passive learning in stream-based scenario.

Fig 8 depicts the classification performance figures of the SSAL, AL and PL methods in the stream-based scenario. As it can be seen, the SSAL method outperforms both the AL and the PL approaches. In particular, for the same number of human labeled instances (6,000 instances), SSAL leads to a 10.0% increase in UAR up to statistic significance in relation to AL with *p*-value = 0.0446 for two-sample Kolmogorov-Smirnov test. Moreover, it reaches the best performance of PL (68.5%) with less 52.2% human effort (i.e., using only 5,500 labeled instances).

In Table 9, we summarize the best performances in a statistically significant way for all methods evaluated (SSAL, AL, and PL) in the pool-based and stream-based scenarios, as well as the number of human-labeled instances needed to achieve that performance. Specifically, in each learning iteration, AL and AL phase of SSAL in both scenarios are all parameterized with a selection of 500 instances for human annotation, the SSL phase of pool-based SSAL selects a number of instances with confidence scores higher than 0.95 for machine annotation, and the SSL phase of stream-based SSAL selects 100 instances with highest confidence scores for machine annotation. As it can be observed, the SSAL effectively reduces the human labeling effort.

**Table 9:** Best performances up to statistic significance achieved using semi-supervised active learning (SSAL), active learning (AL), and passive learning (PL) in pool-based and stream-based scenarios, as well as the number of human-labeled instances (#HLI) needed to achieve that performance.

**Pool-based scenario**			
**Learning methods**	**SSAL**	**AL**	**PL**
**Best UAR (%)**	69.4	69.3	68.5
**#HLI**	6,500	7,500	11,500
**Stream-based scenario**			
**Learning methods**	**SSAL**	**AL**	**PL**
**Best UAR (%)**	68.7	68.7	68.5
**#HLI**	6,000	7,000	11,500

## Conclusion

In this paper, we proposed to tandem Active Learning and Self-Training with the aim of bridging the gap between the desire of sufficient amounts of training data and the scarcity of labeled data in the context of sound classification. In this method, we exploited human and machine labeling with the goal of minimizing the human labeling effort: humans were asked to selectively label those instances that the machine was most uncertain about, and the machine automatically labeled those instances that it could predict with a high confidence level. In order to evaluate the certainty of the labels predicted by the machine annotator, we used a classifier confidence score to determine the informativeness of the labeled instances, which, as demonstrated is a good indicator of the classifier’s certainty about the classification results.

Our proposed method was evaluated on a database with 16,930 instances in both pool-based and stream-based scenarios. Furthermore, we compared our method to Active Learning, Self-Training and Passive Learning. Results show that Active Learning requires significantly less human-labeled data compared to Passive Learning to achieve the same UAR, and that Semi-Supervised Active Learning outperforms both these methods in terms of classification performance and number of human labeled instances necessary to achieve such performance. In both of the pool-based and stream-based scenarios, the Semi-Supervised Active Learning approach allowed us to reduce by 52.2% the amount of human annotations necessary to achieve the best performance of all other methods tested.

While demonstrating the effectiveness of our method, it became also evident that for a successful application of Semi-Supervised Active Learning, the tuning of the confidence threshold is crucial. As we have shown, performance deterioration can occur due to the inclusion of noisy machine-labeled data in the training set. Also, if too many instances are machine-labeled, the classifier performance may never reach a satisfactory level given that very few instances are left for human labeling (considered to be more reliable). Therefore, an optimization process for searching an appropriate threshold is fundamental for the application of Semi-Supervised Active Learning. This tuning is certainly task-specific as it will depend on the complexity of the classification problem (and respective confidence levels), and the objectivity of the ground truth or golden standard (which affects the quality of the labels). While the current fixed threshold strategy may not be suitable in other classification tasks, one can refer to [47], [48] and the references therein for more sophisticated thresholding and selection criteria that delicately balance the trade-off between asking for human labeling versus receiving machine labels.

Finally, and while in this paper we demonstrated the effectiveness of Semi-Supervised Active Learning in largely reducing the need for human annotations in the context of sound classification. Given the non task-specific nature of the algorithm proposed, our method can also be applied to other classification scenarios. In particular, this methodology fits applications in hybrid learning environments where the machine is required to continuously increase and adapt its knowledge about the acoustic environment as well as being able to learn in cooperation with humans.

## References

[1] Phan H, Hertel L, Maass M, Mazur R, Mertins A. Audio phrases for audio event recognition. In: Proc. European Signal Processing Conference. Nice, France; 2015. p. 2591–2595.

[2] Salamon J, Bello JP. Unsupervised feature learning for urban sound classification In: IEEE Int. Conf. Acoustics, Speech, and Signal Processing; 2015.

[3] Ye J, Kobayashi T, Murakawa M, Higuchi T. Robust acoustic feature extraction for sound classification based on noise reduction In: IEEE Int. Conf. Acoustics, Speech, and Signal Processing; 2014 p. 5944–5948.

[4] Wang JC, Lin CH, Chen BW, Tsai MK. Gabor-Based Nonuniform Scale-Frequency Map for Environmental Sound Classification in Home Automation. IEEE Transactions on Automation Science & Engineering. 2014;11(2):607–613. 10.1109/TASE.2013.2285131

[5] Valenzise G, Gerosa L, Tagliasacchi M, Antonacci F, Sarti A. Scream and gunshot detection and localization for audio-surveillance systems In: Proc. IEEE Conf. Advanced Video and Signal Based Surveillance. London, UK; 2007 p. 21—26.

[6] Foggia P, Petkov N, Saggese A, Strisciuglio N, Vento M. Reliable detection of audio events in highly noisy environments. Pattern Recognition Letters. 2015;65:22–28. 10.1016/j.patrec.2015.06.026

[7] Ferguson BG, Lo KW. Acoustic cueing for surveillance and security applications In: Defense and Security Symposium. Orlando, Florida, USA; 2006.

[8] Litvak D, Zigel Y, Gannot I. Fall detection of elderly through floor vibrations and sound In: Proc. IEEE Int. Conf. Engineering in Medicine and Biology Society. Vancouver BC, Canada; 2008 p. 4632—4635. 10.1109/IEMBS.2008.4650245 19163748

[9] Peng YT, Lin CY, Sun MT, Tsai KC. Healthcare audio event classification using iidden Markov models and hierarchical hidden Markov models In: Proc. IEEE Int. Conf. Multimedia and Expo. New York, USA; 2009 p. 1218—1221.

[10] Jin F, Sattar F, Goh DYT. New approaches for spectro-temporal feature extraction with applications to respiratory sound classification. Neurocomputing. 2014;123:362–371. 10.1016/j.neucom.2013.07.033

[11] Dat TH, Li H. Probabilistic distance SVM with Hellinger-Exponential Kernel for sound event classification In: Proc. IEEE Int. Conf. Acoustics, Speech, and Signal Processing. Prague, Czech Republic; 2011 p. 2272–2275.

[12] Fleury A, Noury N, Vacher M, Glasson H, Seri JF. Sound and speech detection and classification in a health smart home In: Proc. IEEE Int. Conf. Engineering in Medicine and Biology Society; 2008 p. 4644–4647. 10.1109/IEMBS.2008.4650248 19163751

[13] Duan S, Zhang J, Roe P, Towsey M. A survey of tagging techniques for music, speech and environmental sound. Artificial Intelligence Review. 2012;42(4):637–661.

[14] Phuong NC, Dat TD. Sound classification for event detection: Application into medical telemonitoring In: Proc. Int. Conf. Computing, Management and Telecommunications (ComManTel); 2013 p. 330–333.

[15] Piczak KJ. ESC: Dataset for Environmental Sound Classification In: Proc. ACM Int. Conf. Multimedia; 2015 p. 1015–1018.

[16] Settles B. Active learning literature survey. University of Wisconsin, Madison; 2010 Available from: http://burrsettles.com/pub/settles.activelearning.pdf.

[17] Riccardi G, Hakkani-Tur D. Active learning: theory and applications to automatic speech recognition. IEEE Trans Audio, Speech, and Language Processing. 2005;13(4):504–511. 10.1109/TSA.2005.848882

[18] Wang M, Hua XS. Active learning in multimedia annotation and retrieval: A survey. ACM Trans Intelligent Systems and Technology. 2011;2(2). 10.1145/1899412.1899414

[19] Zhang Z, Schuller B. Active learning by sparse instance tracking and classifier confidence in acoustic emotion recognition In: Proc. INTERSPEECH. Portland, Oregon, USA; 2012.

[20] Seung HS, Opper M, Sompolinsky H. Query by committee. In: Proc. the 5th Annual Workshop on Computational Learning Theory. Pittsburgh, Pennsylvania, United States; 1992. p. 287–294.

[21] Cohn D, Atlas L, Ladner R. Improving generalization with active learning. Machine Learning. 1994;15(2):201–221. 10.1023/A:1022673506211

[22] Lewis DD, Gale WA. A sequential algorithm for training text classifiers In: Proc. Int. ACM SIGIR Conf. Research and Development in Information Retrieval. Dublin, Ireland; 1994 p. 3–12.

[23] Roma G, Janer J, Herrera P. Active learning of custom sound taxonomies in unstructured audio data In: Proc. Int. Conf. Multimedia Retrieval; 2012 p. 1–2.

[24] Burbidge R, Rowland J, King R. Active learning for regression based on query by committee In: Conf. Intelligent Data Engineering and Automated Learning—IDEAL 2007. vol. 4881 of Lecture Notes in Computer Science. Springer Berlin / Heidelberg; 2007 p. 209–218.

[25] Roy N, McCallum A. Toward optimal active learning through sampling estimation of error reduction In: Proc. 18th Int. Conf. Machine Learning. Williams College, Massachusetts, USA; 2001 p. 441–448.

[26] Yarowsky D. Unsupervised word sense disambiguation rivaling supervised methods. In: Proc. 33rd Annual Meeting Association for Computational Linguistics. Cambridge, Massachusetts; 1995. p. 189–196.

[27] Blum A, Mitchell T. Combining labeled and unlabeled data with co-training In: Proc. 11th Annual Conf. Computational Learning Theory. Madison, Wisconsin, United States; 1998 p. 92–100.

[28] de Sa VR. Learning classification with unlabeled data. Advances in Neural Information Processing Systems. 1994;6:112–119.

[29] Chapelle O, Schölkopf B, Zien A. Semi-supervised learning. Cambridge, MA: MIT Press; 2006.

[30] Zhu X. Semi-supervised learning literature survey. Madison, WI: Department of Computer Sciences, University of Wisconsin at Madison; 2006. TR 1530.

[31] Tur G, Hakkani-Tür D, Schapire RE. Combining active and semi-supervised learning for spoken language understanding. Speech Communication. 2005;45(2):171–186. 10.1016/j.specom.2004.08.002

[32] Zhu X, Lafferty J, Ghahramani Z. Combining active learning and semi-supervised learning using Gaussian fields and harmonic functions. In: Proc. Int. Conf. Machine Learning Workshop on The Continuum from Labelled to Unlabelled Data. Washington DC; 2003. p. 58–65.

[33] Zhang Z, Schuller B. Semi-supervised learning helps in sound event classification In: Proc. 37th IEEE Int. Conf. Acoustics, Speech, and Signal Processing. Kyoto, Japan; 2012 p. 25–30.

[34] Muslea I, Minton S, Knoblock CA. Active + Semi-supervised learning = Robust multi-view learning In: Proc. 19th Int. Conf. Machine Learning. Sydney, Australia; 2002 p. 435–442.

[35] Cui X, Huang J, Chien JT. Multi-view and multi-objective semi-supervised learning for HMM-based automatic speech recognition. IEEE Transactions on Audio, Speech, and Language Processing. 2012;20(7):1923–1935. 10.1109/TASL.2012.2191955

[36] McCallum AK, Nigam K. Employing EM and pool-based active learning for text classification In: Proc. 15th Int. Conf. Machine Learning. Madison, Wisconsin, USA,; 1998 p. 350–358.

[37] Zhou Z, Chen K, Jiang Y. Exploiting unlabeled data in content-based image retrieval In: Proc. 15th European Conf. Machine Learning. Pisa, Italy; 2004 p. 525–536.

[38] Tomanek K, Hahn U. Semi-supervised active learning for sequence labeling. In: Proc. Annual Meeting of the ACL and Int. Joint Conf. Natural Language Processing of the AFNLP. Suntec, Singapore; 2009. p. 1039–1047.

[39] Schuller B, Valstar M, Eyben F, Cowie R, Pantic M. AVEC 2012—The Continuous Audio/Visual Emotion Challenge. In: Proc. Int. Audio/Visual Emotion Challenge and Workshop, Grand Challenge and Satellite of ACM ICMI 2012. Santa Monica, CA; 2012.

[40] Eyben F, Wöllmer M, Schuller B. openSMILE—The Munich Versatile and Fast Open-Source Audio Feature Extractor In: Pro. Int. Conf. ACM Multimedia. Firenze, Italy; 2010 p. 1459–1462.

[41] Hall M, Frank E, Holmes G, Pfahringer B, Reutemann P, Witten IH. The WEKA data mining software: an update. SIGKDD Explor Newsl. 2009 11;11(1):10–18. 10.1145/1656274.1656278

[42] Chawla NV, Bowyer KW, Hall LO, Kegelmeyer WP. SMOTE: Synthetic Minority Over-sampling Technique. Journal of Artificial Intelligence Research. 2002;16:321–357.

[43] Li M, Sethi I. Confidence-based active learning. IEEE Trans Pattern Anal Mach Intell. 2006;28(8):1251–61. 10.1109/TPAMI.2006.156 16886861

[44] Platt J. Probabilistic outputs for support vector machines and comparisons to regularized likelihood methods In: Smola A, Bartlett P, Schölkopf B, Schuurmans D, editors. Advances in large margin classifiers. Cambridge, MA: MIT Press; 1999 p. 61–74.

[45] Duda RO, Hart PE, Stork DG. Pattern classification. 2nd ed New York, NY: John Wiley & Sons; 2001.

[46] Tong S, Koller D. Support vector machine active learning with applications to text classification. Journal of Machine Learning Research. 2002;2(1):45–66.

[47] Beygelzimer A, Dasgupta S, Langford J. Importance weighted active learning In: Proc. Int. Conf. Machine Learning; 2008 p. 49–56.

[48] Nowak RD. Noisy generalized binary search. Advances in Neural Information Processing Systems. 2009;57(12):1366–1374.

